# Medical Examinations in England

**Published:** 1846-07

**Authors:** 


*Medical Examinations in England.*—The London Medical Gazette, for March 6, 1846, contains a Report of the number of candidates examined in Great Britain for Diplomas and Licenses in Medicine and Surgery, and the number of such Diplomas or Licenses actually granted.

It appears that during the three years 1842–'3–'4, the Royal College of Physicians examined 161 candidates, and granted 160 licenses, the rejections being 0·62 per cent.

The Royal College of Surgeons examined 1776 candidates and granted 1576 diplomas; the rejections being 1·12 per cent.

The Society of Apothecaries examined 1137 candidates, and granted 953 certificates, the rejections being 16·1 per cent.

The Univerrsity of London examined 114 (first examination) and passed 72, the rejections being 36·8 per cent. On the second examination, the examined were to the passed as 67 to 60, the rejections being 10·4 per cent.

The University of Cambridge granted diplomas to 10 persons, and licensed 9.

Oxford University examined 6 for diplomas, and granted it to them all. Of eight candidates for license 7 passed.

The number of persons licensed to practice amounted, in England and Wales, in three years, to 2,721, viz: 1,576 surgeons, 953 apothecaries, and 192 physicians, being an annual average of 907 licensed practitioners.—*Ibid.*

